# Weak outcome predictors of multimodal rehabilitation at one-year follow-up in patients with chronic pain—a practice based evidence study from two SQRP centres

**DOI:** 10.1186/s12891-016-1346-7

**Published:** 2016-11-25

**Authors:** Björn Gerdle, Peter Molander, Gunilla Stenberg, Britt-Marie Stålnacke, Paul Enthoven

**Affiliations:** 1 Pain and Rehabilitation Centre, and Department of Medical and Health Sciences, Linköping University, SE-581 85 Linköping, Sweden; 2 Department of Behavioural Sciences and Learning, Linköping University, Linköping, Sweden; 3 Department of Community Medicine and Rehabilitation, Physiotherapy, Umeå University, Umeå, Sweden; 4 Department of Medical and Health Sciences, Faculty of Medicine and Health Sciences, Linköping University, Linköping, Sweden; 5 Department of Community Medicine and Rehabilitation, Rehabilitation Medicine, Umeå University, Umeå, Sweden

**Keywords:** Multidisciplinary, Multimodal, Outcome, Pain, Rehabilitation

## Abstract

**Background:**

For patients with chronic pain, the heterogeneity of clinical presentations makes it difficult to identify patients who would benefit from multimodal rehabilitation programs (MMRP). Yet, there is limited knowledge regarding the predictors of MMRP’s outcomes. This study identifies predictors of outcome of MMRPs at a 12-month follow-up (FU-12) based on data from the Swedish Quality Registry for Pain Rehabilitation (SQRP).

**Methods:**

Patients with chronic pain from two clinical departments in Sweden completed the SQRP questionnaires—background, pain characteristics, psychological symptoms, function, activity/participation, health and quality of life—on three occasions: 1) during their first visit; 2) immediately after the completion of their MMRP; and 3) 12 months after completing the MMRP (*n* = 227). During the FU-12, the patients also retrospectively reported their global impressions of any changes in their perception of pain and their ability to handle their life situation in general.

**Results:**

Significant improvements were found for pain, psychological symptoms, activity/participation, health, and quality of life aspects with low/medium strong effects.

A general pattern was observed from the analyses of the changes from baseline to FU-12; the largest improvements in outcomes were significantly associated with poor situations according to their respective baseline scores. Although significant regressors of the investigated outcomes were found, the significant predictors were weak and explained a minor part of the variation in outcomes (15–25%). At the FU-12, 53.6% of the patients reported that their pain had decreased and 80.1% reported that their life situation in general had improved. These improvements were associated with high education, low pain intensity, high health level, and work importance (only pain perception). The explained variations were low (9–11%).

**Conclusions:**

Representing patients in real-world clinical settings, this study confirmed systematic reviews that outcomes of MMRP are associated with broad positive effects. A mix of background and baseline variables influenced the outcomes investigated, but the explained variations in outcomes were low. There is still a need to develop standardized and relatively simple outcomes that can be used to evaluate MMRP in trials, in clinical evaluations at group level, and for individual patients.

**Electronic supplementary material:**

The online version of this article (doi:10.1186/s12891-016-1346-7) contains supplementary material, which is available to authorized users.

## Background

Approximately 20% of the adult population lives with at least *moderate* chronic pain [1]. Patients with chronic pain describe wide consequences such as intense and disturbing pain, psychological distress and insomnia, reduced work ability and sick-leave, ill-health, and low quality of life [2–6]. According to Years Lived with Disability (YLDs), a measure of non-fatal health outcomes, pain conditions caused 21% of all YLDs globally ahead of 287 other conditions [7]. The five leading conditions of YLDs in Sweden were low back pain, major depressive disorder, falls, neck pain, and other musculoskeletal disorders.

Acute and chronic pain are influenced by and interact with physical, psychological, social, and contextual factors [8–10], thus a bio-psycho-social framework should be considered in clinical practice [11, 12]. Multimodal (i.e., multidisciplinary) rehabilitation programs (MMRPs) are complex interventions based on such a framework and usually continue over several weeks with both general and patient specific goals. Common components of MMRPs are education, supervised physical activity, training in simulated environments, cognitive behavioural therapy (CBT), and work-related efforts. These components can act independently and interdependently resulting in a combination of effects explained by known and unknown mechanisms and these effects are intended to be greater than the sum of its components [13, 14]. The components are coordinated and delivered by a team of different professionals, but an important prerequisite is that the patient is an active participant [15–18].

MMRPs represent important evidence-based progress in treatment for patients with chronic pain [16–21]. Although there is some evidence that MMRPs are effective, the effect sizes are generally small to moderate. Randomized controlled trials (RCT) and systematic reviews (SR) may reflect artificial situations with regard to unrepresentative selection of patients, risk for researcher allegiance, and problems with practical applications of the interventions investigated [22, 23]. Thus, a complementary necessary step is to investigate whether the evidence reported from RCTs and SRs also holds for a consecutive non-selected flow of patients in real-world practice settings. This research methodology—*practice based evidence (PBE)—*is based on prospective observational cohort study designs and has also been applied in rehabilitation research [24].

Patients with chronic pain exhibit heterogeneous clinical presentations and the question arises if certain patient characteristics are indicative of outcomes after MMRP. Answering these questions are important as MMRPs are time consuming and expensive even when most of the activities for the patients are group based. Little is known about outcome predictors of MMRPs [18, 25–30]. It is likely that a combination of predictor variables can be identified [30]. Some of the available predictive studies are based on RCTs, but these RCTs have a small sample of patients and may have limited relevance to MMRP in clinical practice [31–33]. Prediction studies based on clinical samples are needed to reliably identify who actually benefits from inclusion and participation in MMRPs.

This PBE study identifies predictors of outcome of MMRPs for patients with chronic pain based on data from the Swedish Quality Registry for Pain Rehabilitation (SQRP) (www.ucr.uu.se/nrs/) for two multidisciplinary pain centres in Sweden. Six outcome domains were identified (pain intensity, physical functioning, emotional functioning, coping, health and ratings of global improvements) mainly according to the recommendations of the IMMPACT-group [12, 34].

## Methods

### Subjects

This study collected data using the SQRP on 464 patients between 18 and 65 years old who had chronic mainly musculoskeletal pain. These patients were referred to the Pain and Rehabilitation Centre at University Hospital of Linköping or the Pain Rehabilitation Clinic at University Hospital of Umeå between 2008 and 2012. In total, 464 patients with chronic pain were referred to these two facilities to participate in a MMRP. The present study is primarily based on patients who answered the SQRP both at baseline (before the MMRP) and at the 12-month follow-up (*n* = 227).

### MMRP at the two sites

At the Umeå site, the referred patients were assessed by teams consisting of a specialist physician in rehabilitation medicine or by specialists in training under supervision of a senior colleague, a physiotherapist, a social worker, and a psychologist and occupational therapist if needed. If the team considered that the patient needed MMRP and fulfilled the inclusion criteria (disabling chronic pain, on sick leave or experiencing major interference in daily life, no further investigations needed) they were accepted to participate in the program. The MMRP was conducted in groups of six to nine participants and was based on CBT principles and included physiotherapy, ergonomics, training in coping strategies, and education in pain management. The patients were encouraged to take an active part in goal setting. At the Linköping site, medical assessments were performed by senior physicians, primarily from specialists in rehabilitation medicine or similar specialities, or by specialists in training under the supervision of a senior colleague. The majority of the participants were also assessed by a psychologist, an occupational therapist, and a physiotherapist. The Linköping program followed the same main principles as the Umeå program—group treatment from a CBT perspective, group physiotherapy, interventions targeting improved ergonomics, and occupational therapy. At both sites the MMRPs lasted between 6 and 8 weeks for at least 20 h per week of group-based activities. In addition, lectures in basic pain science and pain management were offered for both patients (both sites) as well as for relatives, friends, and colleagues (only Linköping). Both programs included work related advice and support, and individually tailored sessions with team members were available. Further individual sessions might also be required for a few weeks following the program.

The following inclusion criteria for the MMRP were used: (i) disabling chronic pain (on sick leave or experiencing major interference in daily life due to chronic pain); (ii) age between 18 and 65 years; (iii) no further medical investigations needed; (iv) written consent to participate and attend to the MMRP;(v) and agreement not to participate in other parallel treatments. The following exclusion criteria were used: (i) ongoing major somatic or psychiatric disease; (ii) a history of significant substance abuse; and (iii) state of acute crisis. General exclusion criteria included severe psychiatric morbidity, abuse of alcohol and/or drugs, diseases that did not allow physical exercise, and specific pain conditions with other treatments options available (red flags).

### Methods

The SQRP is recognized as a national registry by the Swedish Association of Local Authorities and Regions. The SQRP is mainly based on questionnaires. The patients completed the SQRP questionnaires on three occasions: 1) at the assessment during the first visit to the clinical department (*pre-MMRP* or *baseline*); 2) immediately after MMRP (*post-MMRP*); and 3) at the 12-month follow-up (*FU-12*). Demographic data are only collected before MMRP. In the present study, the predictions concerned the FU-12.

The main purpose of the SQRP is to present the results of MMPR at a group level to the participating clinical departments. Based on these data, health care providers and researchers can develop a process that will encourage continued improvement of rehabilitation programs. In a review of the overall quality of approximately 60 nationwide registries, the Boston Consulting Group considered the SQRP as one of the top ten national registries in Sweden [35].

The SQRP includes descriptive variables of the patients’ background, pain characteristics, other symptoms such as depression and anxiety, function, activity/participation, and quality of life. Generally validated Swedish language versions of the instruments were used.

### Data from the SQRP

Listed below are the variables and instruments of the SQRP that this study used. These are also summarized in Additional file 1: Table S1.

#### Background data

- Age (years)
- Gender
- Education (elementary school, upper secondary school, University or college or other). This variable was dichotomized into University vs the other alternatives.
- Country of birth (Sweden, Another Nordic country, Europe except Nordic country, or Country outside Europe). This variable was dichotomized into Nordic countries vs. outside Nordic countries.
- Currently working/studying (Yes or No) denoted *Work/study-now*.
- Three items concerned the attitude about the future. Those working/studying full time were instructed to skip the first two items below; subjects who were seeking a job were instructed to imagine being employed when answering the following items:
  - ○ What do you think it will be like to return to work/studies or to extend working hours if you are not working full time? (Likert scale: 1 (very easy) to 5 (very difficult). This variable was denoted as *Own prognosis-RTW*.
  - ○ When do you expect to be able to return to work or studies or to work longer if you are not working full time? (Likert scale: 1 (immediately) to 5 (never). This variable was denoted as *RTW-when*.
  - ○ How confident are you about your chances to be fully restored? Likert scale: 1 (totally convinced) to 5 (not at all convinced). This variable is denoted as *Chances-restored.*
- What significance does the work have for you in addition to the importance of income? (Five alternatives: 1) Very important, 2) Important, 3) Partially important, 4) Hardly no importance, or 5) No importance). This variable was denoted as *Work-importance.*

#### Characteristics of pain

- How many times have you visited a physician for your pain complaints the previous year? 0–1 times; 2–3 times; or ≥4 times). This variable was dichotomized into ≥4 times vs. the other alternatives and is denoted as *Dr-visits*.
- When did you first experience the pain you are currently troubled by? (days). This variable was denoted as *Pain-duration*.
- If persistent pain exists, how long? (days). This variable was denoted as *Pain-duration-persistent*.
- Average pain intensity the last week (a numeric rating scale 0–10 with numbers for guidance and the end points also had verbal descriptions (0 = no pain and 10 = worst possible pain). This is denoted as *NRS-7days*.
- Using 36 predefined anatomical areas (18 on the front and 18 on the back of the body), the subjects marked where they experienced pain: 1) head/face, 2) neck, 3) shoulder, 4) upper arm, 5) elbow, 6) forearm, 7) hand, 8) anterior aspect of chest, 9) lateral aspect of chest, 10) belly, 11) sexual organs, 12) upper back, 13) low back, 14) hip/gluteal area, 15) thigh, 16) knee, 17) shank, and 18) foot. The number of areas associated with pain were counted (between 0 and 36) and this variable was labelled the Pain Region Index (*PRI*).

#### Multidimensional Pain Inventory (MPI)

The West Haven-Yale Multidimensional Pain Inventory—(WHY) MPI—is a 61-item self-report questionnaire that measures psychosocial, cognitive, and behavioural effects of chronic pain [36, 37]. Part 1 consists of five scales: Pain severity (MPI-Pain-severity); Interference—pain related interference in everyday life (MPI-PainInterfer); Perceived Life Control (MPI-LifeCon); Affective Distress (MPI-Distress); and Social Support—perceived support from a spouse or significant others (MPI-SocSupp). Part 2 was not used in this study. Part 3 measures to what extent the patients engaged in various activities using four scales. These scales can be combined into a composite scale—the General Activity Index (MPI-GAI)—which was used in the present study [38].

#### Hospital Anxiety and Depression Scale (HADS)

The HADS is a self-assessment questionnaire that measures anxiety and depression using 14 items [39]. HADS comprises seven items in each of the depression and anxiety scales (HAD-D—depression and HAD-A—anxiety). The subscale scores range between 0 and 21, with the lower score indicating the least depression and anxiety possible. HADS is frequently used both in clinical practice and in research and has good psychometric characteristics [39, 40]. It is also validated in its Swedish translation [41].

#### The Chronic Pain Acceptance Questionnaire (CPAQ)

CPAQ measures acceptance behaviours and attitudes towards pain. The process of acceptance refers to the concept of coping with pain; this process changes the focus from controlling pain and related psychological experiences to a wider focus that includes being open to pain without trying to alter the experience. Acceptance includes flexible engagement in goal-directed activities, which include pain as a part of the engagement, without struggling to control pain. CPAQ is a 20-item scale with two subscales: activity engagement (score range: 0–66; denoted CPAQ-AE) and pain willingness (score range: 0–54; denoted CPAQ-PW) [42]. All items are rated on a scale from 0 (never true) to 6 (always true). The CPAQ has shown to be reliable and valid both in the English and Swedish versions [42, 43].

#### The Tampa Scale for Kinesiophobia (Tampa)

Tampa measures fear of movement [44]. The items are rated on a four-point Likert scale from “strongly disagree” to “strongly agree”. The total score has a range from 17 to 68; scores higher than 36 for women and higher than 38 for men indicate high pain-related fear [45]. Tampa has shown to be a reliable assessment tool in chronic pain populations [44, 46]. A study including Dutch, Canadian, and Swedish samples with several different pain types demonstrated that the factor structure was stable across pain diagnoses and nationalities [45].

#### Life Satisfaction Questionnaire (LISAT-11)

LISAT-11 captures the patient’s estimations of satisfaction with life as a whole (LISAT-life) as well as satisfaction in ten specific domains [47]. Each item has six possible answers: 1 = very dissatisfying; 2 = dissatisfying; 3 = fairly dissatisfying; 4 = fairly satisfying; 5 = satisfying; and 6 = very satisfying. This study used LISAT-life together with the following specific items: satisfaction with vocation (LISAT-vocation); satisfaction with economy (LISAT-economy); satisfaction with activities of daily living (ADL) (LISAT-ADL); satisfaction with somatic health (LISAT-somhealth); and satisfaction with psychological health (LISAT-psychhealth).

#### The Short Form Health Survey (SF36)

SF36 intends to represent multidimensional health concepts and measurements of the full range of health states, including levels of well-being and personal evaluations of health [48]. The instrument has eight dimensions (reported using a standardized scale from 0 to 100) [48]. In the present study the scale concerning physical functioning (SF36-PF) was selected. Based on the eight scales, a physical summary component (SF36-PSC) and a mental (psychological) summary component (SF36-MSC) are calculated. Hence, this study used these two summary components together with SF36-PF.

#### The European Quality of Life instrument (EQ-5D)

EQ-5D captures a patient’s perceived state of health [49–51]. The first part of the instrument defines five dimensions: mobility, self-care, usual activities, pain/discomfort, and anxiety/depression. Using these five dimensions, the instrument calculates an index (EQ5D-Index). The EQ5D also measures self-estimation of today’s health according to a 100-point scale, a thermometer-like scale (EQ5D-VAS) with defined end points (high values indicate good health and low values indicate bad health).

#### Retrospective ratings of global improvements in pain and in life situation

The patients retrospectively at FU-12 reported their global evaluations of change in pain (labelled *Retro-pain*) and their ability to handle life situation in general (labelled *Retro-life situation*). The Retro-pain item was rated on a five-point Likert scale from markedly increased pain (0) to markedly decreased pain (4). The other item was rated on a five-point Likert scale from markedly worsened (0) to markedly improved (4). In the regressions, these two items were dichotomized (i.e., no change/worse (0) vs. improved (1).

#### Core outcome domains and measures

In order to predict changes in outcomes, we, as far as possible, chose to adopt the recommendations of the IMMPACT group with regard to output domains and core outcome measures for chronic pain trials [12, 34] and the practical use in the systematic review of de Rooij et al. [28]. In addition to these areas, we also defined two extra domains labelled Coping and Health. When selecting the outcome domains we also considered the contents of the programs. Hence, the following six outcome domains and measures were selected as outcomes in the present study:

- Pain intensity aspects
  - Average pain intensity the last week (NRS-7days)
  - Pain severity according to the Multidimensional Pain Inventory (MPI-Pain severity)

- Physical functioning
  - Pain interference according to MPI (MPI-PainInterfer)
  - Physical functioning according to Short form Health Survey (SF36-PF)

- Emotional functioning
  - Depression according to Hospital Anxiety and Depression Scale (HAD-D)
  - Anxiety according to HADS (HAD-A)
  - Distress according to MPI (MPI-Distress)

- Coping
  - Life control according to MPI (MPI-LifeCon)
  - Activity engagement according to Chronic Acceptance Questionnaire (CPAQ-AE)
  - Pain willingness according to CPAQ (CPAQ-PW)
  - Tampa Scale for Kinesiophobia (Tampa)

- Health
  - perceived state of health (single item) according to European Quality of Life Instrument (EQ5D-VAS)
  - perceived state of health (index) according to EQ (EQ5D-index)
  - Satisfaction with life in general (LISAT-life)

- Ratings of global improvement
  - Retrospective rating of global improvement in life situation (Retro-pain)
  - Retrospective rating of global improvement in life situation (Retro-life situation)

### Statistics

All statistics were performed using the statistical package IBM SPSS Statistics (version 20.0) and SIMCA-P+ (version 13.0; Umetrics Inc., Umeå, Sweden); in all tests, a probability of <0.05 (two-tailed) was accepted as the criteria for significance. In the tables and text, the mean value ± one standard deviation (±1SD) of the investigated variables are given.

Effect sizes of the rehabilitation program were calculated using the template developed by Lakens [52]. In this paper, Cohen’s d_av_ was seen as the most appropriate way to present the effects as this uses the average standard deviation of the two measurement points [53]. Roughly, one can interpret the Cohen’s effect sizes using the following criteria: *d* = 0.2 is small, *d* = 0.5 is medium, and *d* = 0.8 is large [54]. Bonferroni correction was used in the detailed analyses over time for the different outcomes. Results were deemed significant at the *p* = 0.00083 level.

Advanced multivariate analyses (MVDA) are essential in PBE studies. Classical statistical methods such as multiple linear regression (MLR) and logistic regression (LR) can quantify the level of relations of individual factors but disregard interrelationships among different factors and thereby ignore system-wide aspects (e.g., when a group of variables correlates with the investigated dependent outcome) [55]. Classical methods assume variable independence when interpreting results [56]. Eriksson and co-workers discussed the risks with considering one separate variable at a time (COST)[57]. They point out that the COST approach has serious drawbacks; the information in multivariate data often remains hidden and there is a risk for spurious results [57]. To handle these drawbacks, we used advanced MVDA (i.e., *Principal Component Analysis*, PCA) for the multivariate correlation analyses of all investigated variables and *Partial Least Square Regression (*PLS) for the multivariate regressions using SIMCA-P+. These techniques do not require normal distribution [58].

PCA was used to extract and display systematic variation in a data matrix. All variables were log transformed before the statistical analyses if necessary. A cross validation technique was used to identify nontrivial components (p). Variables loading on the same component p were correlated, and variables with high loadings but with opposing signs were negatively correlated. Variables with high absolute loadings—i.e., 95% jack-knife uncertainty confidence interval non equal to zero—were considered significant. Hence, the most important variables were those with high absolute loadings. The obtained components are per definition not correlated and are arranged in decreasing order with respect to explained variation. R^2^ describes the *goodness of fit*—the fraction of sum of squares of all the variables explained by a principal component [59]. Q^2^ describes the *goodness of prediction*—the fraction of the total variation of the variables that can be predicted by a principal component using cross validation methods [59]. Outliers were identified using two methods: 1) score plots in combination with Hotelling’s T^2^ and 2) distance to model in X-space. No extreme outliers were detected.

*PLS* was used for the multivariate regression analyses [59]. The VIP variable (variable influence on projection) indicates the relevance of each X-variable pooled over all dimensions and Y-variables—the group of variables that best explain Y. VIP ≥ 1.0 was considered significant if VIP had 95% jack-knife uncertainty confidence interval non equal to zero. The PLS analyses were made in two steps. First, all potential regressor variables were entered. If this regression was significant, regressors with VIP > 0.80 were selected and used in the final PLS. Coefficients (PLS scaled and centred regression coefficients, Coeff) were used to note the direction of the relationship (positive or negative). SQRP uses predetermined rules when handling single missing items of a scale or a subscale; details about this is reported in Additional file 1: Table S6. SIMCA-P+, in contrast to traditional statistical packages e.g. SPSS, uses the NIPALS algorithm when compensating for missing data (i.e. in the present study the scales and subscales of a certain subject).

MLR or LR could have been an alternative, but they assume that the regressor (X) variables are independent. If multicollinearity (i.e., high correlations) occurs among the X-variables, the regression coefficients become unstable and their interpretability breaks down. MLR and LR also assumes that a high subject-to-variables ratio is present (e.g., > 5), an assumption not required for PLS. In fact, PLS can handle subject-to-variables ratios < 1. Moreover, PLS, in contrast to MLR and LR, can handle several Y-variables simultaneously.

## Results

### Drop out analysis

When comparing those who did not and who did complete the questionnaire at FU-12, only one just barely significant difference was found—the pain intensity variable NRS-7days. Those who did not complete the questionnaire had a mean score of 7.02 (SD 1.06) in comparison to those who did (M = 6.61, SD = 1.89) (*t*(437) = 2.09, *p* = 0.04). Hence, no statistically significant differences were found for demographic variables, psychological well-being, quality of life, acceptance, kinesiophobia, general health, or psychological functioning as measured by the SF-36.

### Background data and characteristics of pain

Background data is reported in Table 1. The majority of the patients were women (>80%). A significant proportion (18.5–36.8%) expressed pessimistic views concerning aspects of return to work (RTW) (Table 1).

**Table 1:** Background data for the patients (*n* = 227)

Variable	
Age (years; mean(SD))	38.1 (10.1)
Women	81.6%
Highest Education (University)	24.1%
Country of origin (Nordic countries)	91.8%
Currently work or studies (Work/study-now)	28.9%
Own prognosis-RTW (very difficult)	36.8%
RTW-when (never)	18.5%
Chances-restored (not at all)	32.3%
Work-importance (very important)	43.0%

Own prognosis-RTW = Prognosis of return to work/studies; RTW-when = When return to work/studies; Chances-restored = Chances to be fully restored; Work-importance = Significance of work (except economic)

The number of anatomical regions with pain (PRI) was 14.7 ± 7.9 out of 36 anatomical regions. Mean pain duration was 2550 ± 2609 days; corresponding figures for persistent pain were 1856 ± 2218 days. Health care seeking was prevalent; 58.9% had visited a physician four or more times during the previous year.

### Effects of MMRP

For the selected outcomes and domains, significant improvements were found, except for LISAT-life (Table 2). The effect sizes were small to medium [60] (Table 2). The largest effect sizes were found for coping aspects followed by pain intensity aspects. The area of emotional functioning generally showed small effect sizes (Table 2). Detailed tables including all variables—also including registrations immediately post MMRP—are shown in Additional file 1: Tables S2–S5.

**Table 2:** Selected outcomes according to the chosen domains before MMRP (Pre) and at 12-month follow-up (FU-12)

*Outcome variables* *(n = 227)*	*Pre* Mean (SD)	*FU-12* Mean (SD)	*Statistics*	Cohen’s d_av_[pre to follow-up]
*Pain intensity*				
NRS-7days	6.8 (1.8)	5.7 (2.2)	*	0.51
MPI-Pain-severity	4.3 (0.9)	3.7 (1.3)	*	0.61
*Physical functioning*				
MPI-Paininterfer	4.5 (0.9)	3.9 (1.2)	*	0.57
SF36-PF	56.1 (19.3)	64.9 (19.5)	*	0.45
*Emotional functioning*				
HAD-D	8.2 (4.0)	6.8 (4.3)	*	0.33
HAD-A	8.2 (4.3)	7.3 (4.2)	*	0.22
MPI-Distress	3.5 (1.2)	3.0 (1.3)	*	0.35
*Coping*				
MPI-LifeCon	2.7 (1.1)	3.2 (1.2)	*	0.49
CPAQ-AE	25.8 (10.9)	33.8 (11.9)	*	0.70
CPAQ-PW	22.5 (8.1)	28.0 (7.9)	*	0.69
Tampa	37.6 (8.1)	32.8 (8.0)	*	0.60
*Health*				
EQ5D-index	0.33 (0.31)	0.45 (0.32)	*	0.38
EQ5D-VAS	36.1 (20.1)	49.3 (21.4)	*	0.63
LISAT-life	3.6 (1.3)	3.7 (1.2)	*ns*	0.14
*Ratings of global improvement*				
Retro-pain				
*Improved (%)*		53.6		
*No change (%)*		39.2		
*Worse (%)*		7.2		
Retro-life situation				
*Improved (%)*		80.1		
*No change (%)*		16.9		
*Worse (%)*		3.0		

The selected outcomes were based mainly on the recommendations of IMMPACT. Mean values (SD) together with statistical analyses Pre vs. FU-12 (*p < .00083) and effect sizes (Cohen’s d) are presented. For the ratings of global improvements are reported percentages (%). Retro-pain = Changes in perception of pain; Retro-life situation = changes in life in general. The variables were trichotomized into improved, no change, and worsened (%)

### Predictions of changes in outcomes at 12-month follow-up

The difference (i.e., changes) between pre and FU-12 values for the selected outcomes were calculated and transformed if necessary so that an improvement had a positive value.

### Regression of changes in pain intensity aspects

In the regression of changes in the two pain intensity aspects (*R* ^2^ = 0.25, *Q* ^2^ = 0.16), the strongest regressors among the background and baseline variables were NRS-7days, MPI-Pain-severity, MPI-SocSupp, Work/study-now, and LISAT-psychhealth (Table 3, model 1). Hence, improvements in pain intensity aspects were associated with the following background and baseline variables: high pain intensities, perceiving social support, working/studying, and satisfaction with psychological health. When excluding the pain intensity variables at baseline, the explained variation decreased as expected (Table 3, model 2). In addition, education (relative high level), low satisfaction with somatic health (LISAT11-somhealth), and relatively short persistent pain duration to some extent were then associated with improvements in pain intensity aspects.

**Table 3:** Regressions of changes in pain intensity, psychological distress, physical functioning and health (Model 1). In model 2 are excluded the baseline variable used for calculating the changes

Model 1			Model 1			Model 1			Model 1		
Changes in pain intensity (*n* = 227)	*VIP*	Sign of Coeff	Changes in psychological distress (*n* = 227)	*VIP*	Sign of Coeff	*Changes in physical functioning* *(n = 227)*	*VIP*	Sign of Coeff	Changes in health aspects (*n* = 202)	*VIP*	Sign of Coeff
NRS-7days	1.74	+	HAD-A	1.49	+	*SF36-PF*	1.73	-	EQ5D-VAS	2.01	-
MPI- pain-severity	1.27	+	HAD-D	1.24	+	*RTW-when*	1.36	-	EQ5D-index	1.52	-
MPI – social support	1.24	+	MPI-Distress	1.24	+	*Own prognosis-RTW*	1.27	-			
Work/study-now	1.14	+	SF36-MCS	1.09	-						
LISAT –psychhealth	1.09	+									
*R* ^*2*^	*0.25*		*R* ^*2*^	*0.15*		*R* ^*2*^	*0.17*		*R* ^*2*^	*0.20*	
*Q* ^*2*^	*0.16*		*Q* ^*2*^	*0.12*		*Q* ^*2*^	*0.13*		*Q* ^*2*^	*0.12*	
Model 2			Model 2			Model 2			Model 2		
Changes in pain intensity (*n* = 227)	*VIP*	Sign of Coeff	Changes in psychological distress (*n* = 227)	*VIP*	Sign of Coeff	*Changes in physical functioning* *(n = 227)*	*VIP*	Sign of Coeff	Changes in health aspects (*n* = 197)	*VIP*	Sign of Coeff
MPI- SocSupp	1.36	+	SF36-MCS	1.38	-	*RTW-when*	1.48	-	Pain-duration-persistent	1.21	-
Work/study-now	1.30	+	MPI-LifeCon	1.10	-	*Own prognosis-RTW*	1.38	-	Age	1.09	-
LISAT-psychhealth	1.22	+	LISAT-life	1.02	-	*SF36-PSC*	1.07	-	Pain duration	1.06	-
Education	1.08	+							RTW-when	1.04	-
LISAT-somhealth	1.01	-							Own prognosis-RTW	1.03	-
Pain-duration-persistent	1.01	-							CPAQ-AE	1.02	-
*R* ^*2*^	*0.14*		*R* ^*2*^	*0.07*		*R* ^*2*^	*0.15*		*R* ^*2*^	*0.05*	
*Q* ^*2*^	*0.10*		*Q* ^*2*^	*0.06*		*Q* ^*2*^	*0.06*		*Q* ^*2*^	*0.03*	

Background and baseline variables (cf. Additional file 1: Table S1) were used as regressors. The significant variables are shown and for each of these are reported VIP (VIP > 1.0 is significant) and the sign of the coefficient (+ or -). The two bottom rows of each model report R^2^ and Q^2^

### Regression of changes in emotional functioning

In the regression of changes in three psychological variables (MPI-Distress, HAD-D, and HAD-A) (*R* ^2^ = 0.14, *Q* ^2^ = 0.10), the strongest regressors of the background and baseline variables were HAD-A, HAD-D, MPI-Distress, and SF36-MCS (Table 3, model 1). Hence, improvements in psychological status were associated with high psychological and mental distress at baseline. When the three psychological variables at baseline were omitted as regressors, the explained variation decreased (Table 3, model 2). In addition, low life control (MPI-LifeCon) and low satisfaction with life in general (LISAT-life) to some extent was associated with improvements in the three psychological variables.

### Regression of changes in physical functioning

In the regression of changes in physical functioning (i.e., MPI-PainInterfer and SF36-PF; *R* ^2^ = 0.19, *Q* ^2^ = 0.13), the three significant regressors were SF36-PF, RTW-when, and Own prognosis-RTW (Table 3, model 1). Hence, improvements in physical functioning were associated with the following circumstances at baseline: low physical functioning (SF36-PF), perceptions of relatively soon return to work (i.e., RTW-when), and relatively easy to return to work (i.e., Own prognoisis—RTW). When excluding the two physical functioning variables at baseline as regressors, the explained variation decreased (Table 3, model 2). To some extent low physical health (SF36-PSC) at baseline was associated with improvements in physical functioning.

### Regression of changes in coping aspects

It was not possible to significantly regress the changes in the four coping aspects (i.e., the 4 Y-variables: MPI-LifeCon, CPAQ-AE, CPAQ-PW, and Tampa) using the background and baseline variables as regressors.

### Regression of changes in health aspects

In the significant regression of changes in the health variables (i.e., the two EQ5D variables and LISAT-life; *R* ^2^ = 0.19, *Q* ^2^ = 0.09), the two significant regressors were EQ5D-VAS and EQ5D-index (Table 3, model 1). Hence, improvements in health aspects were associated with low reported health at baseline.

When omitting the health variables at baseline, the explained variation decreased (Table 3, model 2). Short pain durations and low age were then associated with improvements in health aspects.

### Retrospective evaluations of pain and in ability to handle life situation in general

At FU-12, 53.6% retrospectively reported improvements for pain and 80.1% for general life situation (Table 2). For Retro-pain, the following background and baseline variables were multivariately important (*R* ^2^ = 0.11, *Q* ^2^ = 0.04): Education (VIP = 1.84 +), MPI-Pain severity (VIP = 1.42-), EQ5D-index (VIP = 1.07+), and Work-importance (VIP = 1.07-). Hence, improvements were associated with relatively high education, low pain intensity (MPI-pain-severity), high health (EQ-5D-index), and perceiving work as important for reasons other than economic (i.e. Work- importance). For Retro-life situation, the important variables at baseline (*R* ^2^ = 0.09, *Q* ^2^ = 0.05) were MPI-Pain severity (VIP = 1.77-), EQ5D-index (VIP = 1.72+), NRS-7days (VIP = 1.25-), and education (VIP = 1.20+). Hence, improvements in life situation were associated with low pain intensities (NRS-7days and MPI-Pain-severity), high health (EQ5D-index), and relatively high education at baseline.

### PCA of outcome variables

To understand to what extent the changes in outcomes of the different domains together with the two retrospective items above were independent, a PCA was made for the situation at FU-12. Only one significant component (*R* ^2^ = 0.34; *Q* ^2^ = 0.24) was achieved according to the cross-validation rules in SIMCA-P+. The four most important variables – i.e., highest absolute loadings—were changes in MPI-PainInterfer (loading = 0.33), MPI-LifeCon (loading = 0.31), MPI-Pain severity (loading = 0.30), and MPI-Distress (loading = 0.29). Hence, these four variables showed the most prominent variability across subjects.

### Regression of changes in the four MPI variables

When regressing the changes in the four most important MPI variables according to the PCA, the most important background/baseline variables were MPI-LifeCon (VIP = 2.05-), MPI-Distress (VIP = 1.46+), and Work–importance (VIP = 1.08-)(*R* ^2^ = 0.16, *Q* ^2^ = 0.09, *n* = 227). Hence, improvements in these four MPI variables were associated at baseline with low life control, high psychological distress, and perceiving work as important for reasons other than economic.

## Discussion

Major results of the present study are listed below.

- The majority of subjects reported benefits of participating in MMRP according both to the repeated measurements (i.e., changes from baseline to FU-12) and to the retrospective evaluations.
- Pain intensity and emotional aspects showed the strongest improvements (i.e., effect sizes) according to the repeated measurements.
- The analyses of the repeated measurements showed that the largest improvements in outcomes were significantly associated with poor situations according to their respective baseline scores.
- Although significant regressors of the investigated domains of outcomes were found, the significant predictors were weak and only explained a small part of the variation in outcomes.

### Effectiveness of MMRP

This PBE study confirms the generally positive results for MMRPs according to SRs [16–18], health economics studies, and 10-year follow-ups [19–21] (Table 2). Both the repeated measurements (i.e., baseline vs. FU-12) and the retrospective items (Table 2) showed improvements for this cohort of patients.

### Retrospective evaluations at FU-12

The majority of patients retrospectively reported improvements according to both pain and in their ability to handle their life situation in general (Table 2). Fortunately and in agreement with other studies [61, 62], relatively small proportions (3–7%) of the patients reported worse situations at FU-12 (Table 2). Qualitative explorations have been suggested as one way to develop interventions for patients who do not benefit from MMRP [63].

A mix of background and baseline factors were important regressors. The two retrospective items had several regressors in common. That is, low pain intensity, high education, and high health perception were related to improvements. Similar results have been reported in other studies using retrospective evaluations [33, 61]. In contrast, a SR of MMRP for fibromyalgia patients summarized that the global treatment effect was predicted by the MPI dysfunctional profile (i.e., high pain, disability, and high solicitous responses from significant others) and/or worse baseline status [28]. Hence, it is presently unclear if a relatively good or bad situation with respect to, for example, pain intensity is associated with improvements when evaluated retrospectively.

### Changes in outcomes according to the repeated measurements

In the repeated measurements (i.e., changes from baseline to FU-12), the most prominent effects according to Cohen’s d were for emotional and pain aspects (Table 2). Effect sizes of 0.51–0.61 for the two pain intensity variables at FU-12 were found (Table 2) and in fact both variables showed increasing effect sizes over time (Additional file 1: Tables S2 and S4). These results were in contrast to some SRs, which reported no evidence for efficacy with respect to pain intensity [16, 17]. Many pain patients consider pain intensity as the most important aspect to improve in treatments. Several of the included RCTs in SRs of MMRP do not include pain intensity due to the fact that the interventions are not focussed on the pain itself but rather on its consequences [16, 17]. Some authors have suggested that it might be detrimental for the patient to focus on attempting to control, reduce, or cure pain, since this might shift emphasis away from the aspects that are important for the patient’s health and quality of life, such as daily functioning and emotional well-being [64, 65]. Although these lines of arguments appear reasonable, it may ethically problematic if both clinical practice and research ignore the reports of the patients regarding pain intensity. Moreover, patients with high pain intensities require greater reductions in pain intensity than patients with lower pain intensity levels in order to obtain clinically important improvements [66]. In addition to focusing on pain intensity, it might be wise to focus on the other outcomes emphasised by IMMPACT when communicating with patients. As an outcome of MMRP, pain intensity might be associated with problems when interpreting the results of MMRPs (e.g., a successful intervention leads to less fear of movement) as the patient may be more prone to participate in strenuous activities, which may lead to increased pain intensity in a more active patient. However, changes in pain intensity, psychological distress, and coping aspects were positively intercorrelated in this study, findings that do not support such an argument. It is presently unclear if a more prominent focus on pain intensity within the bio-psycho-social context of MMRP can increase the effect sizes or will actually diminish them.

A general pattern was observed where changes in outcomes were significantly predicted by their respective baseline scores (Table 3, model 1). For example, high pain intensity at baseline was associated with more prominent changes in pain intensity aspects. Such results have been observed in other studies and could be due to the fact that a more severe initial status gives more room for improvement [61]. Another related explanation could be regression to the mean [33]. Recently, Bonstra et al. briefly summarized the literature concerning regressors of repeated measures of outcomes and better outcomes were reported for patients with high scores on depression, high fear avoidance beliefs, high perceived stress, optimistic attitudes, low need to socialize, younger age, or high educational level [61]. Hence, the present results (Table 3, model 1) and a study of chronic widespread pain generally agree with that review [33]. In contrast to this literature, a SR concerning fibromyalgia reported that poor outcomes were associated with depression at baseline [28]. Differences across studies with respect to important predictors may be due to factors such as number of potential prognostic factors, cohort characteristics, chosen outcomes, and/or content of MMRP [18, 29, 67, 68].

For two of the domains of outcomes—pain intensity and physical functioning (Table 3, model 1)—additional background and baseline variables were significant. Hence, the regression of the pain intensity changes was also important for satisfaction with psychological health, working/studying, and relatively high social support at baseline. For changes in physical functioning, additional significant regressors were optimistic attitudes towards RTW (i.e., RTW-when and Own-prognosis–RTW).

Based on the regressions of the changes in the repeated measurements (Table 3, model 1), it seems important to choose patients with a relatively severe clinical picture in order to be able to achieve substantial improvements. It is reasonable to assume that this is relevant only up to a certain level of severity. However, even though it was possible to significantly regress most of the domains of outcomes, the explained variations (*R* ^2^) were low (15–25%). Similar results have been reported by other researchers [61]. Hence, other—not investigated—factors are important and the present results concerning significant background and baseline variables cannot be applied directly in clinical practice with any precision.

### Is high or low pain intensity indicative of positive outcomes?

The importance of the level of pain intensity for positive outcome differed between the repeated measurements and the retrospective evaluations. Improvements in pain intensity aspects according to the repeated measures were associated with *high* pain intensity at baseline (Table 3, model 1), while in the retrospective evaluations the improvements were associated with *low* pain intensity at baseline. There are several problems with retrospective items such as recall time, “telescoping”, desirability, and memory aspects [69–71]. When using retrospective items to evaluate changes in a treatment context, additional problems may exist (e.g., overly optimistic assessments) [72]. Hence, the results concerning predictors for the retrospective items may have been associated with bias. On the other hand, repeated evaluations using self-report questionnaires in intervention studies may be problematic [73]. The change that the patient undergoes because of the intervention may affect the interpretations of the questions when presented at follow-ups. MMRPs aim to influence the perception and coping of pain, psychological distress, and various behaviours. Hence, there may be a risk that the attitude towards the items of the questionnaire changed as a consequence of MMRP. This could mean that the follow-up tests cannot simply be compared with the test before MMRP. In order to deepen the knowledge concerning efficacy of MMRPs, objective registrations (e.g., sick-leave registrations, actigraphic recordings, and qualitative interviews [63]) can be important additional sources.

### Evaluations of outcomes of MMRP

There is a need to develop clinically applicable and standardized ways to evaluate multiple outcomes of MMPRs in individual patients in trials and in PBE studies. This involves both the statistical methods (cf. Statistics) and strategies for handling multiple outcomes. This study used different approaches for evaluation of outcomes. The number of outcomes in MMRPs are generally high; one SR including 46 RCTs found in median nine outcomes and that these generally were not divided into primary and secondary outcomes [16]. In a recent SR, the outcomes were evaluated separately [74], which may be problematic if the outcomes are intercorrelated and in fact they often are as found in the present study. The present study used 14 outcomes divided into six domains that were mainly recommendations from IMMPACT [12, 28, 34]. Different definitions of a positive outcome of a MMRP trial have been presented such as the *majority of outcomes* had to be significantly better than for the control intervention [16, 17]. The authors of another SR predetermined *primary* and *secondary outcomes* and what was necessary to classify an intervention as positive before reviewing the RCTs [75].

The analysis of several outcomes may raise an issue of multiple comparisons and Bonferroni corrections are frequently applied [76] and recommended in pain trials [77]. However, this is a conservative approach when the number of tests increases [76, 78, 79], and this approach reduces the chances to detect real treatment effects. Furthermore, such corrections were designed for corrections of *independent* comparisons [78]. The latter is generally not present when evaluating the outcome of MMRPs. IMMPACT has presented hierarchical or “gatekeeping” procedures that do not require adjustment for multiplicity [77] but a natural hierarchy of the outcomes. Outcomes may be combined into a single composite outcome [80], but this may be problematic with respect to missing cases and when the components of the composite endpoint are measured on different scales such as non-commensurate outcomes [80].

Multivariate methods that are able to handle non-commensurate outcomes in one analysis have been presented [80]. The presently applied PLS regression can also handle non-commensurate outcomes in one analysis. We used a more comprehensive approach when regressing four outcomes simultaneously. These four outcomes were chosen based on the advanced multivariate correlation analysis (PCA). In agreement with the regressions of the repeated measures for different domains, we found that a relatively severe clinical presentation at baseline was associated with improvements.

### Limitations

There are several limitations to consider. No control group was included and outcomes can be due both to the effects of MMRP and to other factors such as natural course. On the other hand, these patients on average had their pain for a considerable period (on average more than 2500 days), a period that reduces but does not eliminate the risk that effects are related to the natural course. Patients participating in MMRPs have often tried different unimodal treatments with little or no effects and thus represent a selection of patients. The present cohort of patients also represents a selection of patients with the most complicated clinical pictures as they were referred to a university hospital. Hence, the generalizability of the present results is limited and the results of MMRP in primary care may be different. Not all the MMRP participants answered the questionnaire at FU-12. The only significant difference at baseline was a small difference in pain intensity. But bias cannot be ruled out since the non-responders may have had worse effects of MMRP than the responders. It was not possible to analyse our results in relation to the contents and it was not possible to control for small changes in content over time. There are no internationally accepted definitions of MMRP [61, 81] and no taxonomies for the contents of these programs exist [17, 61], both issues that hinder further improvements. The general goals of MMRP may not be relevant to—or seen as relevant by—every patient. In the present study, the importance of the general goals was not investigated and handled in the regressions. Another limitation is that we have no records regarding the level of patient attendance on the programs.

## Conclusion

This study—representing the consecutive non-selected flow of patients in real-world clinical practice settings using SQRP—confirmed systematic reviews that outcomes of MMRP are associated with broad positive effects. A mix of background and baseline variables affected the outcomes investigated, but the explained variations in outcomes were low. In the future there is a need to develop a standardized and relatively simple outcome that can be used to evaluate a complex intervention such as MMRP in trials, in clinical evaluations at group level, and for individual patients.

## Additional file

Additional file 1: Table S1. Demographic and outcome variables before MMRP (denoted Pre) and 12-month follow-up (FU-12) used in the study. **Table S2.** Comparison of results on pain, psychological variables and QoL. **Table S3.** Comparison of results on the SF-36 at pre, post, and follow-up. **Table S4.** Comparison of results on MPI at pre, post, and follow-up. **Table S5.** Comparison of results on LISAT at pre, post, and follow-up. **Table S6.** Handling of single missing items of the different instruments. (DOCX 29 kb)

## Abbreviations

CBT: Cognitive behavioural therapy
CPAQ: Chronic Pain Acceptance Questionnaire
CPAQ-AE: The Chronic Pain Acceptance Questionnaire subscale activity engagement
CPAQ-PW: The Chronic Pain Acceptance Questionnaire subscale pain willingness
Dr-visits: Number of visits to a physician previous year
EQ5D-index: The European Quality of Life instrument index
EQ5D-VAS: The European Quality of Life instrument health scale
FU-12: 12-month follow-up
HADS: Hospital Anxiety and Depression Scale
HADS-A: Hospital Anxiety and Depression Scale subscale anxiety
HADS-D: Hospital Anxiety and Depression Scale subscale depression
ICF: International Classification of Functioning, Disability and Health
LISAT: Life Satisfaction Questionnaire
LISAT-ADL: The Life Satisfaction Questionnaire subscale Activity of daily living
LISAT-psychhealth: The Life Satisfaction Questionnaire subscale psychological health
LISAT-somhealth: The Life Satisfaction Questionnaire subscale somatic health
LR: Logistic regression
MLR: Multiple linear regression
MMRP: Multimodal rehabilitation programs
MPI: Multidimensional Pain inventory
MPI-GAI: The MPI subscale General Activity Index
MPI-LifeCon: the MPI subscale Perceived Life Control
MPI-PainInterfer: The MPI subscale pain related interference in everyday life
MPI-SocSupp: The MPI subscale Social support
MVDA: Advanced multivariate analyses
NRS-7days: Average pain intensity the last week
PBE: practice based evidence
PLS: Partial Least Square Regression
PRI: Pain region index
RCT: Randomized controlled trials
Retro-life situation: Retrospective global evaluation of change in ability to handle life situation in general
Retro-pain: Retrospective global evaluation of change in pain
RTW: Return to work
SD: Standard deviation
SF36: The Short Form Health Survey
SF36-MSC: The Short Form Health Survey mental summary component
SF36-PF: The Short Form Health Survey subscale physical functioning
SF36-PSC: The Short Form Health Survey physical summary component
SQRP: Swedish Quality Registry for Pain Rehabilitation
SR: Systematic reviews
Tampa: The Tampa Scale for Kinesiophobia
YLD: Years Lived with Disability

## References

[1] Breivik H, Collett B, Ventafridda V, Cohen R, Gallacher D (2006). Survey of chronic pain in Europe: prevalence, impact on daily life, and treatment. Eur J Pain.

[2] Turk DC, Dworkin RH, Revicki D, Harding G, Burke LB, Cella D, Cleeland CS, Cowan P, Farrar JT, Hertz S (2008). Identifying important outcome domains for chronic pain clinical trials: an IMMPACT survey of people with pain. Pain.

[3] Casarett D, Karlawish J, Sankar P, Hirschman K, Asch DA (2001). Designing pain research from the patient’s perspective: what trial end points are important to patients with chronic pain?. Pain Med.

[4] Robinson ME, Brown JL, George SZ, Edwards PS, Atchison JW, Hirsh AT, Waxenberg LB, Wittmer V, Fillingim RB (2005). Multidimensional success criteria and expectations for treatment of chronic pain: the patient perspective. Pain Med.

[5] Brown JL, Edwards PS, Atchison JW, Lafayette-Lucey A, Wittmer VT, Robinson ME (2008). Defining patient-centered, multidimensional success criteria for treatment of chronic spine pain. Pain Med.

[6] Merrick D, Sundelin G, Stålnacke BM (2012). One-Year Follow-up of Two Different Rehabilitation Strategies for Patients with Chronic Pain. J Rehabil Med.

[7] Vos T, Flaxman AD, Naghavi M, Lozano R, Michaud C, Ezzati M, Shibuya K, Salomon JA, Abdalla S, Aboyans V (2012). Years lived with disability (YLDs) for 1160 sequelae of 289 diseases and injuries 1990-2010: a systematic analysis for the Global Burden of Disease Study 2010. Lancet.

[8] Linton S, Bergbom S (2011). Understanding the link between depression and pain. Scand J Pain.

[9] Ossipov MH, Dussor GO, Porreca F (2010). Central modulation of pain. J Clin Invest.

[10] Gatchel R, Peng Y, Peters M, Fuchs P, Turk D (2007). The biopsychosocial approach to chronic pain: scientific advances and future directions. Psychol Bull.

[11] WHO (2001). International Classification of Functioning, Disability and Health (ICF).

[12] Dworkin RH, Turk DC, Farrar JT, Haythornthwaite JA, Jensen MP, Katz NP, Kerns RD, Stucki G, Allen RR, Bellamy N (2005). Core outcome measures for chronic pain clinical trials: IMMPACT recommendations. Pain.

[13] Hawe P, Shiell A, Riley T (2004). Complex interventions: how “out of control” can a randomised controlled trial be?. BMJ.

[14] Campbell M, Fitzpatrick R, Haines A, Kinmonth AL, Sandercock P, Spiegelhalter D, Tyrer P (2000). Framework for design and evaluation of complex interventions to improve health. BMJ.

[15] Bennett M, Closs S (2010). Methodological issues in nonpharamacological trials for chronic pain. Pain Clin Updates.

[16] SBU (2006). Methods for treatment of chronic pain a systematic review of the literature (In Swedish: Metoder för behandling av långvarig smärta : en systematisk litteraturöversikt).

[17] SBU (2010). Rehabilitation of chronic pain [In Swedish: Rehabilitering vid långvarig smärta. En systematisk litteraturöversikt]. SBU-rapport.

[18] Scascighini L, Toma V, Dober-Spielmann S, Sprott H (2008). Multidisciplinary treatment for chronic pain: a systematic review of interventions and outcomes. Rheumatology.

[19] Busch H, Bodin L, Bergstrom G, Jensen IB (2011). Patterns of sickness absence a decade after pain-related multidisciplinary rehabilitation. Pain.

[20] Norrefalk JR, Ekholm K, Linder J, Borg K, Ekholm J (2008). Evaluation of a multiprofessional rehabilitation programme for persistent musculoskeletal-related pain: economic benefits of return to work. J Rehabil Med.

[21] Jensen IB, Busch H, Bodin L, Hagberg J, Nygren A, Bergstrom G (2009). Cost effectiveness of two rehabilitation programmes for neck and back pain patients: A seven year follow-up. Pain.

[22] Munder T, Brutsch O, Leonhart R, Gerger H, Barth J (2013). Researcher allegiance in psychotherapy outcome research: an overview of reviews. Clin Psychol Rev.

[23] Margison FR, Barkham M, Evans C, McGrath G, Clark JM, Audin K, Connell J (2000). Measurement and psychotherapy. Evidence-based practice and practice-based evidence. Br J Psychiatry.

[24] Whiteneck GG, Gassaway J (2013). SCIRehab uses practice-based evidence methodology to associate patient and treatment characteristics with outcomes. Arch Phys Med Rehabil.

[25] Ahlberg M, Axelsson S, Eckerlund I, Gerdle B, Stålnacke B-M, Söderlund A, Åsenlöf P, Andersson G: Rehabilitering vid långvarig smärta: En systematisk litterturöversikt. In: Stockholm: SBU - Statens beredning för medicinisk utvärdering; 2010.

[26] Laisné F, Lecomte C, Corbière M (2012). Biopsychosocial predictors of prognosis in musculoskeletal disorders: a systematic review of the literature (corrected and republished). Disabil Rehabil.

[27] Heiskanen T, Roine R, Kalso E (2012). Multidisciplinary pain treatment - which patients do benefit?. Scand J Pain.

[28] de Rooij A, Roorda LD, Otten RH, van der Leeden M, Dekker J, Steultjens MP (2013). Predictors of multidisciplinary treatment outcome in fibromyalgia:a systematic review. Disabil Rehabil.

[29] van der Hulst M, Vollenbroek-Hutten MM, Ijzerman MJ (2005). A systematic review of sociodemographic, physical, and psychological predictors of multidisciplinary rehabilitation-or, back school treatment outcome in patients with chronic low back pain. Spine (Phila Pa 1976).

[30] Laisne F, Lecomte C, Corbiere M (2012). Biopsychosocial predictors of prognosis in musculoskeletal disorders: a systematic review of the literature (corrected and republished) *. Disabil Rehabil.

[31] Ang DC, Bair MJ, Damush TM, Wu J, Tu W, Kroenke K (2010). Predictors of pain outcomes in patients with chronic musculoskeletal pain co-morbid with depression: results from a randomized controlled trial. Pain Med.

[32] Oh TH, Hoskin TL, Luedtke CA, Weingarten TN, Vincent A, Kim CH, Thompson JM (2012). Predictors of clinical outcome in fibromyalgia after a brief interdisciplinary fibromyalgia treatment program: single center experience. PM R.

[33] de Rooij A, van der Leeden M, Roorda LD, Steultjens MP, Dekker J (2013). Predictors of outcome of multidisciplinary treatment in chronic widespread pain: an observational study. BMC Musculoskelet Disord.

[34] Turk DC, Dworkin RH, Allen RR, Bellamy N, Brandenburg N, Carr DB, Cleeland C, Dionne R, Farrar JT, Galer BS (2003). Core outcome domains for chronic pain clinical trials: IMMPACT recommendations. Pain.

[35] Nationell satsning på kvalitetsregister inom vården (In Swedish) [www.forskasverige.se/wp-content/uploads/Nationell-Satsning-Kvalitetsregister.pdf]

[36] Turk DC, Rudy TE (1988). Toward an empirically derived taxonomy of chronic pain patients: integration of psychological assessment data. J Consult Clin Psychol.

[37] Turk DC, Rudy TE (1987). Towards a comprehensive assessment of chronic pain patients. Behav Res Ther.

[38] Bergström G, Jensen IB, Bodin L, Linton SJ, Nygren AL, Carlsson SG (1998). Reliability and factor structure of the Multidimensional Pain Inventory--Swedish Language Version (MPI-S). Pain.

[39] Zigmond AS, Snaith RP (1983). The hospital anxiety and depression scale. Acta Psychiatr Scand.

[40] Bjelland I, Dahl AA, Haug TT, Neckelmann D (2002). The validity of the Hospital Anxiety and Depression Scale. An updated literature review. J Psychosom Res.

[41] Lisspers J, Nygren A, Söderman E (1997). Hospital Anxiety and Depression Scale (HAD): some psychometric data for a Swedish sample. Acta Psychiatr Scand.

[42] McCracken L, Vowles K, Eccleston C (2004). Acceptance of chronic pain: component analysis and a revised assessment method. Pain.

[43] Wicksell R, Olsson G, Melin L (2008). The Chronic Pain Acceptance Questionnaire (CPAQ)-further validation including a confirmatory factor analysis and a comparison with the Tampa Scale of Kinesiophobia. Eur J Pain.

[44] Vlaeyen J, Kole-Snijders A, Boeren R, van Eek H (1995). Fear of movement/(re)injury in chronic low back pain and its relation to behavioral performance. Pain.

[45] Roelofs J, Sluiter J, Frings-Dresen M, Goossens M, Thibault P, Boersma K, Vlaeyen J (2007). Fear of movement and (re)injury in chronic musculoskeletal pain: Evidence for an invariant two-factor model of the Tampa Scale for Kinesiophobia across pain diagnoses and Dutch, Swedish, and Canadian samples. Pain.

[46] Crombez G, Vlaeyen J, Heuts P, Lysens R (1999). Pain-related fear is more disabling than pain itself: evidence on the role of pain-related fear in chronic back pain disability. Pain.

[47] Fugl-Meyer AR, Fugl-Meyer KS (1988). The coping process after traumatic brain injury. Scand J Rehabil Med Suppl.

[48] Sullivan M, Karlsson J, Ware J (1995). The Swedish 36 Health survey. Evaluation of data quality, scaling assumption, reliability and construct validity across general populations in Sweden. Soc Sci Med.

[49] EuroQol (1990). EuroQol: a new facility for the measurement of health-related quality of life. Health Policy.

[50] Brooks R (1996). EuroQol: the current state of play. Health Policy.

[51] Dolan P, Sutton M (1997). Mapping visual analogue scale health state valuations onto standard gamble and time trade-off values. Soc Sci Med.

[52] Lakens D: Calculating and reporting effect sizes to facilitate cumulative science: a practical primer for t-tests and ANOVAs. *Frontiers in Psychology* 2013, 863(4(November),):doi:10.3389/fpsyg.2013.00863. 10.3389/fpsyg.2013.00863 PMC3840331 24324449

[53] Cumming G (2012). Understanding the New Statistics: Effect sizes, Confidence Intervals, and Meta-Analysis.

[54] Cohen J (1988). Statistical Power Analysis for the Behavioral Sciences.

[55] Jansen JJ, Szymanska E, Hoefsloot HC, Jacobs DM, Strassburg K, Smilde AK (2012). Between Metabolite Relationships: an essential aspect of metabolic change. Metabolomics.

[56] Pohjanen E, Thysell E, Jonsson P, Eklund C, Silfver A, Carlsson IB, Lundgren K, Moritz T, Svensson MB, Antti H (2007). A multivariate screening strategy for investigating metabolic effects of strenuous physical exercise in human serum. J Proteome Res.

[57] Eriksson L, Byrne T, Johansson E, Trygg J, Vikström C (2013). Multi- and Megavariate Data Analysis - Basic Principles and Applications.

[58] Wheelock AM, Wheelock CE (2013). Trials and tribulations of ’omics data analysis: assessing quality of SIMCA-based multivariate models using examples from pulmonary medicine. Mol Biosyst.

[59] Eriksson L, Johansson E, Kettaneh-Wold N, Trygg J, Wikström C, Wold S (2006). Multi- and Megavariate Data analysis; part I and II.

[60] Cohen J (1992). A power primer. Psychol Bull.

[61] Boonstra AM, Reneman MF, Waaksma BR, Schiphorst Preuper HR, Stewart RE (2015). Predictors of multidisciplinary treatment outcome in patients with chronic musculoskeletal pain. Disabil Rehabil.

[62] Morley S, Williams A, Hussain S (2008). Estimating the clinical effectiveness of cognitive behavioural therapy in the clinic: evaluation of a CBT informed pain management programme. Pain.

[63] Matthias MS, Miech EJ, Myers LJ, Sargent C, Bair MJ (2012). “There’s more to this pain than just pain”: how patients’ understanding of pain evolved during a randomized controlled trial for chronic pain. J Pain.

[64] Thompson M, McCracken LM (2011). Acceptance and related processes in adjustment to chronic pain. Curr Pain Headache Rep.

[65] McCracken LM, Zhao-O’Brien J (2010). General psychological acceptance and chronic pain: There is more to accept than the pain itself. Eur J Pain.

[66] Salaffi F, Stancati A, Silvestri CA, Ciapetti A, Grassi W (2004). Minimal clinically important changes in chronic musculoskeletal pain intensity measured on a numerical rating scale. Eur J Pain.

[67] Michaelson P, Sjolander P, Johansson H (2004). Factors predicting pain reduction in chronic back and neck pain after multimodal treatment. Clin J Pain.

[68] Keel PJ, Wittig R, Deutschmann R, Diethelm U, Knusel O, Loschmann C, Matathia R, Rudolf T, Spring H (1998). Effectiveness of in-patient rehabilitation for sub-chronic and chronic low back pain by an integrative group treatment program (Swiss Multicentre Study). Scand J Rehabil Med.

[69] Pina-Sánchez J, Koskinen J, Plewis I (2012). Measurement Error in retrospective reports of unemployment. *CCSR Working paper.* The Cathie Marsh Centre for Census and Survey Research.

[70] Bernard H, Killworth P, Kronenfeld D, Sailer L (1984). The problem of informant accuracy: the validity of retrospective data. Ann Rev Anthropol.

[71] Van der Vaart W, Van der Zouwen J, Dijkstra W (1995). Retrospective questions: data quality, task difficulty and the use of checklist. Qual Quantity.

[72] Schwartz N, Stone A, Shiffman S, Atienza A, Nebeling L (2007). Retrospective and concurrent self-reports: the rationale for real-time data capture. The science of real-time data capture: Self-reports in health research.

[73] Westlander G (2004). Refined use of standardized self-reporting in intervention studies (In swedish: Förfinad användning av standardiserad självrapportering i interventionstudier). Socialvetenskaplig tidskrift.

[74] Kamper SJ, Apeldoorn AT, Chiarotto A, Smeets RJ, Ostelo RW, Guzman J, van Tulder MW (2014). Multidisciplinary biopsychosocial rehabilitation for chronic low back pain. Cochrane Database Syst Rev.

[75] Scascighini L, Toma V, Dober-Spielmann S, Sprott H (2008). Multidisciplinary treatment for chronic pain: a systematic review of interventions and outcomes. Rheumatology (Oxford).

[76] Feise RJ (2002). Do multiple outcome measures require p-value adjustment?. BMC Med Res Methodol.

[77] Turk DC, Dworkin RH, McDermott MP, Bellamy N, Burke LB, Chandler JM, Cleeland CS, Cowan P, Dimitrova R, Farrar JT (2008). Analyzing multiple endpoints in clinical trials of pain treatments: IMMPACT recommendations. Initiative on Methods, Measurement, and Pain Assessment in Clinical Trials. Pain.

[78] Bagiella E (2009). Clinical trials in rehabilitation: single or multiple outcomes?. Arch Phys Med Rehabil.

[79] Tyler KM, Normand SL, Horton NJ (2011). The use and abuse of multiple outcomes in randomized controlled depression trials. Contemp Clin Trials.

[80] Teixeira-Pinto A, Mauri L (2011). Statistical analysis of noncommensurate multiple outcomes. Circ Cardiovasc Qual Outcomes.

[81] Hauser W, Bernardy K, Arnold B, Offenbacher M, Schiltenwolf M (2009). Efficacy of multicomponent treatment in fibromyalgia syndrome: a meta-analysis of randomized controlled clinical trials. Arthritis Rheum.

