# Supplementary material for: Weak outcome predictors of multimodal rehabilitation at one-year follow-up in patients with chronic pain—a practice based evidence study from two SQRP centres
Source: BMC Musculoskelet Disord. 2016 Nov 25;17:490. doi: 10.1186/s12891-016-1346-7 (PMC5124266; doi:10.1186/s12891-016-1346-7)
Supplement: Additional file 1: Table S1. — Demographic and outcome variables before MMRP (denoted Pre) and 12-month follow-up (FU-12) used in the study. Table S2. Comparison of results on pain, psychological variables and QoL. Table S3. Comparison of results on the SF-36 at pre, post, and follow-up. Table S4. Comparison of results on MPI at pre, post, and follow-up. Table S5. Comparison of results on LISAT at pre, post, and follow-up. Table S6. Handling of single missing items of the different instruments. (DOCX 29 kb) [file 12891_2016_1346_MOESM1_ESM.docx]

# **Additional file**

**Table S1**. Demographic and outcome variables before MMRP (denoted Pre) and 12-month follow-up (FU-12) used in the study.

| ***Variables*** | ***Pre*** | ***FU-12*** |
| --- | --- | --- |
| ***Background data*** |  |  |
| Age | **X** |  |
| Gender | **X** |  |
| Education | **X** |  |
| Country of birth | **X** |  |
| Own prognosis-RTW | **X** |  |
| RTW-when | **X** |  |
| Chances-restored | **X** |  |
| Work-importance | **X** |  |
| ***Pain aspects*** |  |  |
| NRS-7days | **X** | **X** |
| PRI | **X** |  |
| Dr-visits | **X** | **X** |
| Pain-duration | **X** |  |
| Pain-duration-persistent | **X** |  |
| MPI-Pain-severity | **X** | **X** |
| MPI-PainInterfer | **X** | **X** |
| ***Psychological aspects*** |  |  |
| HADS-D | **X** | **X** |
| HADS-A | **X** | **X** |
| MPI-Distress | **X** | **X** |
| ***Participation aspects*** |  |  |
| Working/study-now | **X** | **X** |
| MPI-SocSupp | **X** | **X** |
| MPI-GAI | **X** | **X** |
| LISAT-vocation | **X** | **X** |
| LISAT-economy | **X** | **X** |
| LISAT-ADL | **X** | **X** |
| SF36-PF | **X** | **X** |
| ***Coping*** |  |  |
| MPI-LifeCon | **X** | **X** |
| CPAQ-AE | **X** | **X** |
| CPAQ-PW | **X** | **X** |
| Tampa | **X** | **X** |
| ***Health aspects*** |  |  |
| LISAT-life | **X** | **X** |
| LISAT-somhealth | **X** | **X** |
| LISAT-psychhealth | ***X*** | ***X*** |
| SF36-MSC | **X** | **X** |
| SF36-PSC | **X** | **X** |
| EQ5D-index | **X** | **X** |
| EQ5D-VAS | **X** | **X** |
| ***Global rating of MMRP effects*** |  |  |
| Retro-pain |  | **X** |
| Retro-life situation |  | **X** |

**Table S2**. Comparison of results on pain, psychological variables and QoL.

| Variable | **Pre** | **Post (sd)** | **Cohen’s d_av_** **[Pre-Post]** | **t** | **FU-12** | **Cohen’s d_av_ [Pre to FU-12]** | **t** |
| --- | --- | --- | --- | --- | --- | --- | --- |
|  | **(sd)** |  |  | **(df)** |  |  | **(df)** |
| ***Pain*** |  |  |  |  |  |  |  |
| NRS-7days | 6.76 (1.81) | 6.11 (2) | 0.34 | 5.65* (338) | 5.74 (2.2) | 0.51 | 6* (219) |
| ***Psychological aspects*** |  |  |  |  |  |  |  |
| HAD – A | 8.22 (4.28) | 7.38 (4.15) | 0.2 | 4.14* (367) | 7.3 (4.19) | 0.22 | 4.41* (231) |
| HAD – D | 8.15 (3.95) | 6.64 (3.98) | 0.38 | 7.6* (367) | 6.79 (4.34) | 0.33 | 5.3* (231) |
| CPAQ-AE | 25.83 (10.86) | 31.47 (10.79) | 0.52 | -9.76* (231) | 33.76 (11.85) | 0.7 | -7.77* (145) |
| CPAQ-PW | 22.5 (8.07) | 25.56 (7.73) | 0.39 | -6.29* (249) | 27.97 (7.88) | 0.69 | -7.51* (150) |
| Tampa | 37.62 (8.05) | 34.38 (6.85) | 0.44 | 7.82* (241) | 32.79 (7.96) | 0.6 | 7.59* (148) |
| ***QoL*** |  |  |  |  |  |  |  |
| EQ5D-index | 0.33 (0.31) | 0.41 (0.32) | 0.25 | -4.75* (296) | 0.45 (0.32) | 0.38 | -4.91* (173) |
| EQ5D-VAS | 36.11 (20.1) | 47.31 (21.82) | -0.53 | -8.95* (328) | 49.25 (21.38) | -0.63 | -8.96* (207) |

Mean values (SD) together with statistical analyses Pre vs. immediately post MMRP and Pre vs. at FU-12 (t and (df); * *p<* .00083) are reported together with effect sizes (Cohen´s d).

|  |  |
| --- | --- |

**Table S3**. Comparison of results on the SF-36 at pre, post, and follow-up.

| **Variable** | **Pre** | **Post (sd)** | **Cohen’s d_av_ [Pre-Post]** | **t** | **FU-12**  **(sd)** | **Cohen’s d_av_ [Pre to FU-12]** | **t** |
| --- | --- | --- | --- | --- | --- | --- | --- |
|  | **(sd)** |  |  | **(df)** |  |  | **(df)** |
| **SF36-PF** | 56.14 (19.33) | 60.56 (19.02) | 0.23 | -5.28* (300) | 64.92 (19.52) | 0.45 | -6.71* (177) |
| **SF36-RP** | 12.26 (23.94) | 19.48 (29.66) | 0.27 | -3.76* (298) | 25.66 (35.14) | 0.45 | -4.08* (176) |
| **SF36-BP** | 25.21 (14.4) | 33.14 (15.98) | 0.52 | -7.98* (298) | 34.75 (18.22) | 0.59 | 6.11* (176) |
| **SF36-GH** | 41.06 (18.03) | 46 (20.08) | 0.26 | -5.72* (295) | 50.53 (21.78) | 0.48 | -6.14* (176) |
| **SF36-V** | 23.5 (17.47) | 32.67 (21.54) | 0.47 | -7.66* (296) | 32.32 (24.14) | 0.42 | -5.84* (174) |
| **SF36-SF** | 49.33 (24.01) | 52.72 (24.8) | 0.14 | -2.45 NS (298) | 56.43 (27.34) | 0.28 | -3.52* (176) |
| **SF36-RE** | 44.22 (43.49) | 53.46 (42.76) | 0.21 | -3.38* (293) | 56.95 (42.6) | 0.3 | -2.81^NS^ (174) |
| **SF36-MH** | 59.61 (18.79) | 65.43 (18.73) | 0.31 | -5.55* (296) | 64.51 (19.82) | 0.25 | -4.1* (177) |
| **SF36-MSC** | 36.75 (11.34) | 40 (11.58) | 0.28 | -4.78* (284) | 39.78 (12.67) | 0.25 | -3.11* (171) |
| **SF36-PSC** | 29.32 (7.03) | 31.44 (8.4) | 0.28 | -4.79* (284) | 33.67 (9.27) | 0.53 | -5.88* (171) |

Mean values (SD) together with statistical analyses Pre vs. immediately post MMRP and Pre vs. at FU-12 (t and (df); * *p<* .00083, ^ns^ = not significant) are reported together with effect sizes (Cohen´s d).

**Table S4:** Comparison of results on MPI at pre, post, and follow-up.

| **Variable** | **Pre** | **Post**  **(sd)** | **Cohen’s d_av_ [Pre-Post]** | **t** | **FU-12**  **(sd)** | **Cohen’s d_av_ [Pre to FU-12]** | **t** |
| --- | --- | --- | --- | --- | --- | --- | --- |
|  | **(sd)** |  |  | **(df)** |  |  | **(df)** |
| **MPI –Pain-severity** | 4.33 (0.9) | 3.95 (1.06) | 0.39 | 7.41* (364) | 3.67 (1.27) | 0.61 | 8.88* (234) |
| **MPI-Paininterfer** | 4.49 (0.89) | 4.12 (0.99) | 0.39 | 8.52* (365) | 3.89 (1.2) | 0.57 | 9.18* (223) |
| **MPI-LifeCon** | 2.69 (1.05) | 3.21 (1.06) | 0.49 | -8.11* (366) | 3.23 (1.15) | 0.49 | -6.76* (233) |
| **MPI – Distress** | 3.46 (1.15) | 2.95 (1.3) | 0.42 | 7.37* (367) | 3.02 (1.34) | 0.35 | 6.04* (234) |
| **MPI – SocSupp** | 4.23 (1.26) | 3.93 (1.29) | 0.24 | 6.1* (366) | 3.69 (1.25) | 0.43 | 6.74* (234) |
| **MPI – GAI** | 2.4 (0.77) | 2.63 (0.71) | 0.31 | -6.66* (366) | 2.59 (0.81) | 0.24 | -4.02* (234) |

Mean values (SD) together with statistical analyses Pre vs. immediately post MMRP and Pre vs. at FU-12 (t and (df); * *p<* .00083) are reported together with effect sizes (Cohen´s d).

**Table S5**. Comparison of results on LISAT at pre, post, and follow-up.

| Variable | Pre | Post  (sd) | Cohen’s d_av_ [Pre-Post] | t (df) | FU-12  (sd) | Cohen’s d_av_ [Pre to FU-12] | t (df) |
| --- | --- | --- | --- | --- | --- | --- | --- |
| LISAT-life | 3.56 (1.32) | 3.8 (1.1) | 0.2 | -3.18 (374)* | 3.74 (1.2) | 0.14 | -1.4 (236)^ns^ |
| LISAT-vocation | 2.99 (1.54) | 2.81 (1.53) | 0.12 | 2.21 (351)^ns^ | 3.11 (1.53) | 0.08 | -1.08 (226)^ns^ |
| LISAT-economy | 3.34 (1.43) | 2.96 (1.52) | 0.26 | 6.15 (358)* | 3.24 (1.47) | 0.07 | 2.21 (231)^ns^ |
| LISAT-ADL | 4.47 (1.21) | 4.53 (1.25) | 0.05 | 2.03 (337)^ns^ | 4.69 (1.24) | 0.18 | -2.17 (233)^ns^ |
| LISAT-Somhealth | 2.46 (1.15) | 2.65 (1.16) | 0.17 | -2.72 (356)^ns^ | 2.88 (1.27) | 0.35 | -4.75 (231)* |
| LISAT-psychhealth | 3.53 (1.39) | 3.78 (1.26) | 0.19 | -3.29 (382)* | 3.81 (1.36) | 0.2 | -3.35 (240)* |

Mean values (SD) together with statistical analyses Pre vs. immediately post MMRP and Pre vs. at FU-12 (t and (df); * *p<* .00083, ^ns^ = not significant) are reported together with effect sizes (Cohen´s d).

**Table S6:** Handling of single missing items of the different instruments.

| ***Instrument*** | ***Handling*** |
| --- | --- |
| HADS | For each subscale (Depression and anxiety) is accepted 2 missing items. These are replaced with the mean of the answered items for each subscale. |
| MPI | For each subscale with more than 3 items is accepted 2 missing items. These are replaced with the mean of the answered items for each subscale. For subscales with 3 items is accepted 1 missing item. This is replaced with the mean of the answered items. |
| SF36 | At least half of the items of each subscale must be answered. Missing items are then replaced with the mean value of the subscale. For subscales with only two items is not accepted missing items. |
| Tampa | Two missing items are accepted. These are replaced with the mean of the answered items. |
| CPAQ | For each subscale is accepted 2 missing items. These are replaced with the mean of the answered items for each subscale. |
| EQ | No missing items is accepted. |
| LISAT- 11 | Only single items. |
